# Aluminum-Induced Amyloidogenesis and Impairment in the Clearance of Amyloid Peptides from the Central Nervous System in Alzheimer’s Disease

**DOI:** 10.3389/fneur.2014.00167

**Published:** 2014-09-05

**Authors:** Yuhai Zhao, James M. Hill, Surjyadipta Bhattacharjee, Maire E. Percy, Aileen I. D. Pogue, Walter J. Lukiw

**Affiliations:** ^1^LSU Neuroscience Center, Louisiana State University Health Sciences Center, Louisiana State University, New Orleans, LA, USA; ^2^Department of Ophthalmology, Louisiana State University Health Sciences Center, Louisiana State University, New Orleans, LA, USA; ^3^Department of Microbiology, Louisiana State University Health Sciences Center, Louisiana State University, New Orleans, LA, USA; ^4^Department of Physiology, University of Toronto, Toronto, ON, Canada; ^5^Department of Obstetrics and Gynaecology, University of Toronto, Toronto, ON, Canada; ^6^Neurogenetics Laboratory, Surrey Place Centre, Toronto, ON, Canada; ^7^Alchem Biotech, Toronto, ON, Canada; ^8^Department of Neurology, Louisiana State University Health Sciences Center, New Orleans, LA, USA

**Keywords:** amyloidogenesis, Alzheimer’s disease, Aβ42 peptide monomers, phagocytosis, aluminum, TREM2

## Overview

The membrane-integral beta-amyloid precursor protein (βAPP) is probably the most intensively studied brain cell protein in neurobiology. βAPP is processed by tandem beta-gamma secretase cleavage into 42 amino acid amyloid (Aβ42) peptides, whose progressive accumulation is one distinguishing feature of Alzheimer’s disease (AD) neuropathology (1–3). While homeostatic amounts of Aβ42 peptide generation and clearance seem to be tolerated by brain cells, their over-abundance, aggregation into higher order structures, and inability of brain cells to effectively phagocytose and clear these intensely hydrophobic peptides contribute to the pro-inflammatory and neurotoxic pathology of AD. Aluminum, as an extremely high charge density cation (*Z*^2^/*r* = 18), has the remarkable capability to both (1) aggregate and compact Aβ42 peptide monomers into higher order, more neurotoxic oligomeric, and fibrillar structures, and (2) impair, at the molecular-genetic level, the cellular machinery responsible for Aβ42 peptide monomer phagocytosis and clearance from the cell (4–13). This opinion paper will briefly assess these two remarkable, functionally overlapping, and decidedly neurotoxic properties of aluminum: (1) on the ability of physiologically realistic amounts of aluminum to aggregate Aβ42 peptide monomers into higher order dimeric, oligomeric, and fibrillar structures, and (2) on the ability of aluminum to impair at nanomolar concentrations and at the level of epigenetic regulation, microglial cell-mediated clearance mechanisms of Aβ42 peptides from the extracellular space of the brain and CNS.

## Aluminum, Aβ42 Peptides, and Amyloidogenesis

The polytopic transmembrane glycoprotein βAPP (~770 amino acids), highly expressed in human neurons has been implicated as a regulator of neuronal cytoarchitecture, synaptic plasticity, axon guidance, and cell–cell interactions in the brain and retina (1–3). Via tandem beta-gamma secretase cleavage, Aβ42 peptides are naturally generated as monomers from βAPP, however, due to their intensely lipophilic and hydrophobic character (21.4% valine–isoleucine), Aβ42 monomers rapidly aggregate into higher order structures forming Aβ42 dimers, oligomers, and fibrils to ultimately aggregate to form the core of the senile plaque lesions that in part characterize AD (1–3). Factors, which accelerate Aβ42 peptide monomer aggregation, such as aluminum sulfate and other aluminum and metallic salts, apparently do so by cross-linking anionic amino acids contained within the 42 amino acid Aβ42 peptide sequence, to form larger Aβ42 peptide-containing “clumps” (1, 6). These roughly spherical deposits eventually form into dense, insoluble senile plaques ~100 μm or more in diameter that are often visible to the unaided eye after Congo Red or other suitable amyloid staining (2–6). Aβ42 peptide coalescence apparently occurs in much the same way as the aluminum in hydrated aluminum potassium sulfate [alum; KAl(SO_4_)_2_.12H_2_O], which is used worldwide as a clarifying agent in water purification, reacts with impurities. When added to turbid drinking water, aluminum crosslinks organic impurities, allowing them to flocculate or “stick together” and precipitate out of solution, resulting in a highly clarified and esthetically pleasing “finished” water product (14). Interestingly, aggregation of Aβ42 peptides into senile plaques can be visualized in the living brain using multi-photon *in vivo* imaging of transgenic mouse models of AD, and can take as little as 24 h to form, a remarkably rapid time course in light of the fact that AD represents such a slowly progressing neurological disease with age-related amyloid deposition (15). In particular, interactions with biosphere-abundant, AD-relevant metal ions such as aluminum may lead to the formation of highly structured amyloid aggregates with peculiar biophysical properties that are associated with an extremely high degree of neurotoxicity (3–6, 16–20). Aluminum’s stimulatory effects on such AD-type neuropathology are further supported by the observation that transgenic murine models for AD (TgAD), such as the amyloid-overexpressing Tg2576 model, when fed aluminum in their diet, exhibit increased oxidative stress, pro-inflammatory signaling, and even more robust senile plaque lesion formation compared to non-aluminum fed TgAD controls (21, 22).

## Aluminum-Mediated Down-Regulation of the Phagocytosis Sensor Protein TREM2

**Figure 1:**
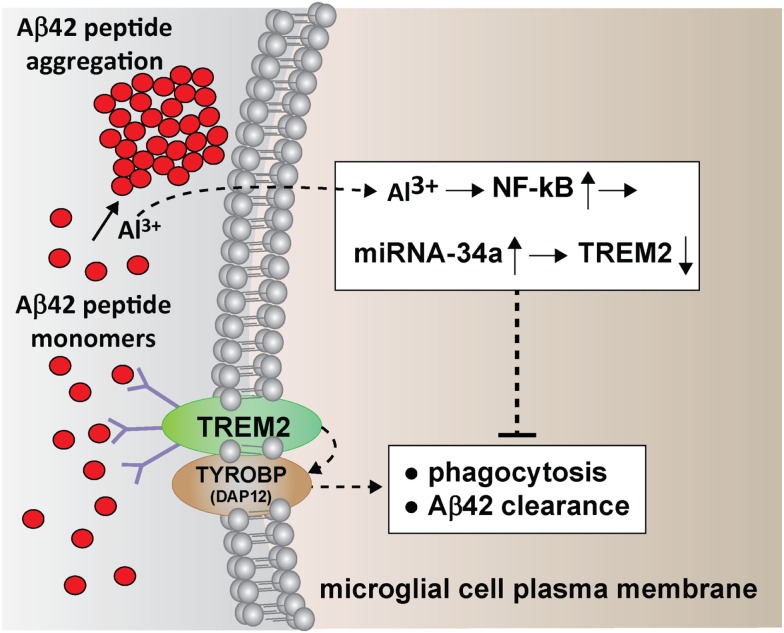
**Multiple neurotoxic actions of aluminum results in an impairment in the clearance of Aβ42 peptides that drives amyloidogenesis and AD-type change**; in intra-cellular and intra-nuclear compartments, aluminum induces NF-kB (5, 14, 15), up-regulates miRNA-34a (9, 10), and down-regulates TREM2, a key microglial intra-membrane phagocytic sensor protein (6–8, 11); lack of sufficient TREM2 impairs microglial cell-mediated phagocytosis and clearance of Aβ42 peptide monomers; deficits in TREM2 (but not the TREM2-associated TYROBP/DAP12 adaptor protein required for phagocytosis and Aβ42 peptide engulfment) have been widely reported in AD brain and in stressed microglial cells (7, 8, 11); in the extracellular space (upper left) aluminum aggregates Aβ42 peptide monomers into dense insoluble spherical clumps and promotes senile plaque formation; the movement of Al3+ across the plasma membrane is not well understood but may involve both active and passive transport; while microglia are able to phagocytose Aβ42 peptide monomers that they may have difficulty with higher order aggregates resulting in microglial activation and a pathogenic pro-inflammatory response that contributes to AD neuropathology.

Active microglial cell-mediated phagocytic processes that remove excessive, neurotoxic, or end-stage molecules from the brain are a naturally occurring mechanism to cleanse the neural environment and maintain neural homeostasis (7–11). Failure of these continuous homeostatic phagocytic processes has potential pro-inflammatory, innate-immune, and neuropathogenic consequences (8–13, 20). Interestingly, microglial cells, the “roving phagocytic scavengers” of the human brain and CNS surround aggregating Aβ42 peptides and when so activated appear to play a role in inflammatory and immune aspects of AD pathogenesis including the further generation of brain cell-damaging reactive oxygen species (ROS) (5, 17–19). One key microglial transmembrane-spanning stimulatory sensor–receptor glycoprotein of the immune-globulin/lectin-like gene superfamily essential for phagocytosis of Aβ42 peptides in the brain and CNS is the inducible triggering receptor expressed in myeloid/microglial cells (TREM2; Figure 1) (7–13). This glycoprotein, an important player in the CNS innate-immune response extends molecular sensors into the extracellular environment to sense and recognize molecular tags located on Aβ42 peptides – this is accomplished by poorly understood molecular-pattern recognition mechanisms (Figure 1) (10–13). Aluminum sulfate, a known stimulator of ROS and the pro-inflammatory transcription factor NF-kB, is also known to induce a small family of pro-inflammatory microRNAs including miRNA-34a (7–12). An up-regulated miRNA-34a in turn down-regulates TREM2 expression in the microglial cell membrane leading to a deficit in the ability of microglial cells to effectively phagocytose Aβ42 peptide monomers (9, 10, 13). This ultimately results in a buildup of Aβ42 peptides in the extracellular space, and thus favors their self-aggregation into higher order amyloid species (Figure 1).

Taken together, these results strongly suggest that one of the most prominent features of AD – the progressive accumulation and aggregation of Aβ42 peptides into senile plaques – is driven by aluminum via multiple interdependent pathogenic mechanisms. These findings continue to support an active role for aluminum in key neuropathogenic, inflammatory, and amyloidogenic pathways that contribute to the AD process. Aluminum appears to therefore drive AD-relevant amyloidogenic pathology (1) directly, by aggregation of Aβ42 peptide monomers into higher order structures that subsequently form into senile plaque cores, and (2) indirectly, through an NF-kB- and miRNA-34a-mediated epigenetic mechanism that contributes to a down-regulation in the expression of the key phagocytosis sensor protein TREM2, and a failure of microglial cells to naturally sense, phagocytose, and eliminate neurotoxic Aβ42 peptides. Hence, both of these pathways have strong potential to contribute, perhaps cooperatively, to the failure to adequately phagocytose naturally generated Aβ42 peptide monomers, thus promoting their aggregation and driving amyloidogenesis with pro-inflammatory consequences. Indeed, the aluminum-mediated aggregation of Aβ42 peptide monomers into higher order structures of higher molecular mass may preclude them from being adequately “ingested” by microglial cell-mediated phagocytic mechanisms, further supporting their accumulation and self-association in the extracellular space. It will certainly be interesting to see if aluminum salts perform similar pathogenic roles in other progressive, age-related neurodegenerative disorders of the CNS with an innate-immune, inflammatory, and/or amyloidogenic component.

## Conflict of Interest Statement

The authors declare that the research was conducted in the absence of any commercial or financial relationships that could be construed as a potential conflict of interest.
